# Effectively infinite optical path-length created using a simple cubic photonic crystal for extreme light trapping

**DOI:** 10.1038/s41598-017-03800-y

**Published:** 2017-06-23

**Authors:** Brian J. Frey, Ping Kuang, Mei-Li Hsieh, Jian-Hua Jiang, Sajeev John, Shawn-Yu Lin

**Affiliations:** 1 Department of Physics, Rensselaer Polytechnic Institute, 110 8th St., Troy, NY 12180 USA; 2 Department of Photonics, National Chiao-Tung University, No. 1001, Daxue Rd, East District, Hsinchu City, 300 Taiwan; 3 School of Physical Science and Technology, Soochow University, 1st Shizi Street, Suzhou, Jiangsu 215006 China; 4 Department of Physics, University of Toronto, 60 Saint George St., Toronto, Ontario M5S 1A7 Canada

## Abstract

A 900 nm thick TiO_2_ simple cubic photonic crystal with lattice constant 450 nm was fabricated and used to experimentally validate a newly-discovered mechanism for extreme light-bending. Absorption enhancement was observed extending 1–2 orders of magnitude over that of a reference TiO_2_ film. Several enhancement peaks in the region from 600–950 nm were identified, which far exceed both the ergodic fundamental limit and the limit based on surface-gratings, with some peaks exceeding 100 times enhancement. These results are attributed to radically sharp refraction where the optical path length approaches infinity due to the Poynting vector lying nearly parallel to the photonic crystal interface. The observed phenomena follow directly from the simple cubic symmetry of the photonic crystal, and can be achieved by integrating the light-trapping architecture into the absorbing volume. These results are not dependent on the material used, and can be applied to any future light trapping applications such as phosphor-converted white light generation, water-splitting, or thin-film solar cells, where increased response in areas of weak absorption is desired.

## Introduction

In several areas of photonics and optoelectronics, the efficient absorption and conversion of light into useful energy is of paramount importance. Currently, the performance of materials otherwise suitable for economical large-scale production is limited by different factors, such as imperfect near-infrared absorption (crystalline silicon^1^ and ruthenium-based dyes^2, 3^), or charge diffusion length (amorphous silicon^4^). To circumvent these limitations while addressing cost and material consumption^5–7^, charge transport^8^ and other efficiency concerns^9, 10^, it is beneficial to engineer structures that can extend the photon’s optical path length by altering how light flows through these devices. This enhances light-matter interaction without using more material. The term most used to describe this behavior is “light trapping”^11–20^.

Historically, the standard for light trapping has involved random scattering from rough surfaces^21^, but periodic structures have garnered interest recently because of their ability to exploit the wave nature of light. Motivated by the question of whether these “ordered” architectures can surpass the light trapping capability of their random counterparts, this strategy has produced a variety of devices, such as those based on distributed Bragg reflectors^14, 16^, Mie scattering^17^, or the guided-modes of a textured dielectric slab^22, 23^. Additionally, some studies have reported structures that are capable of surpassing the ergodic limit in certain frequency ranges^24–26^.

In this work, we identify a precise mechanism for path length enhancement, which has its origin in the refraction of light by a three-dimensional photonic crystal^27, 28^ (PC). This effect, called parallel-to-interface refraction (PIR), extends the path length by orders of magnitude by coupling light into modes for which the Poynting vector lies nearly parallel to the PC interface. PIR has been studied theoretically by John and colleagues^11–13^, but it is a challenge to verify experimentally because it has been tested only on materials for which the base absorption saturates in some part of the spectrum^29^. To accurately measure the performance of any light trapping scheme, it is necessary to use weakly absorbing materials as a reference. This allows one to identify frequency ranges where strong enhancement is obtainable; devices could then be designed to target areas of weak absorption using the well-known scaling properties of PCs.

Accordingly, we overcome this difficulty by constructing a simple cubic PC from high-refractive index (*n* > 2) transparent TiO_2_ to observe orders-of-magnitude enhancement over a reference TiO_2_ film, for which the base absorption is less than 1% across the entire visible-NIR spectrum. The conclusions that will follow from this analysis are not restricted to TiO_2_ or any material in particular. Angular dependence measurements will reveal the structure of the photonic dispersion of our PC and prove that this enhancement is caused by PIR. The experimental validation of PIR provides an opportunity to use simple cubic PCs as an ordered, three-dimensional network that refracts light according to the dispersion relation. This approach to absorption enhancement is amenable to infiltration by various agents, such as dyes, polymers, and nanophosphors, and is also suitable for investigating PC-induced light emission enhancement. We anticipate that the scope of these results will extend beyond thin-film solar cells to other applications, like phosphor conversion in light-emitting diodes^30^, and water-splitting^20^.

## Results

### TIO_2_ Photonic Crystal

**Figure 1:**
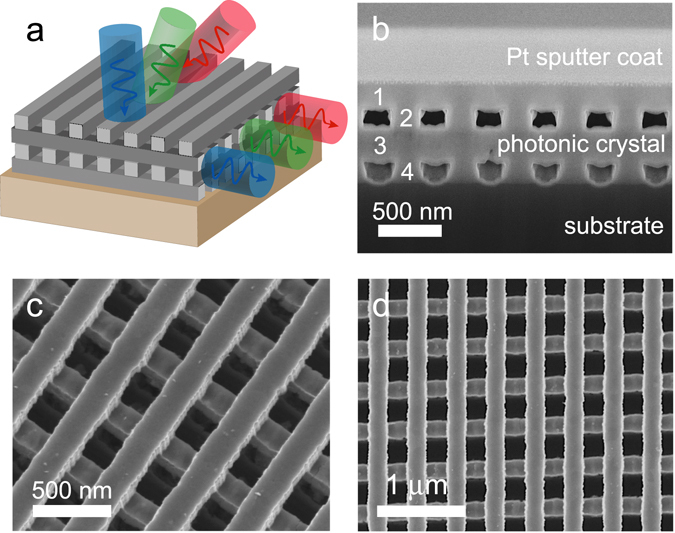
Schematic of a TiO_2_ simple cubic photonic crystal. (**a**) Schematic of TiO_2_ PC depicting parallel-to-interface refraction. (**b**) Cross-section side view (**c**) perspective view and (**d**) top view of fabricated TiO_2_ PC.

The structure used for this study, depicted in Fig. 1a, is a stacked, 4-layer TiO_2_ simple cubic photonic crystal (see Methods section for structural details). A complete discussion of the fabrication of this structure is recorded in a previous work^31^, but the results are summarized in the scanning electron micrographs of Fig. 1b–d. In addition to a schematic of the PC, Fig. 1a further depicts the bending of light via parallel-to-interface refraction. In this simple example, light of different frequencies is incident on the PC from air, at different angles relative to the surface normal. Inside the PC, the energy carried by the wave travels at the group velocity along a direction parallel to the PC/air interface, before exiting through the sidewall of the PC. This is simply an example to help visualize how light would propagate under PIR. In practice, the PC’s lateral dimensions are semi-infinite in extent. Thus, PIR ideally provides photons with an effectively infinite path length through the PC, a result that manifests as a spike in the absorption spectrum at the resonant frequency where the necessary boundary conditions are satisfied. We now discuss these conditions in detail.

### Origin of Parallel-To-Interface Refraction

**Figure 2:**
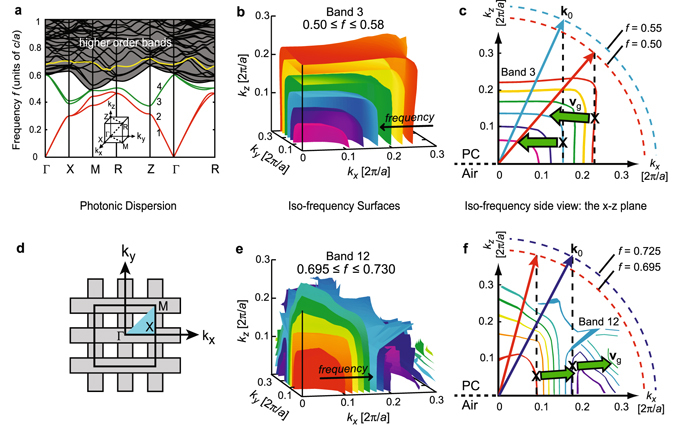
Bandstructure and iso-frequency surfaces of a simple cubic photonic crystal. (**a**) Photonic bandstructure of the simple cubic PC. The normalized frequency *f* is plotted in units of *c*/*a* where *c* is the speed of light in vacuum and *a* is the lattice constant. The inset shows the first Brillouin zone of the simple cubic lattice. (**b**) Iso-frequency surfaces for frequencies in band 3. (**c**) Cross-section of the band 3 iso-frequency surfaces demonstrating PIR. (**d**) Schematic of the top surface of the PC, with the *x-y* plane of the BZ overlaid to show the geometry of the problem. (**e**) Iso-frequency surfaces for frequencies in band 12. (**f**) Cross-section of the band 12 surfaces demonstrating PIR.

The origin of PIR in simple cubic PCs may be found through investigation of the photonic bandstructure, which is shown in Fig. 2a. The frequency *f* is plotted on the *y*-axis in units of *c*/*a*, where *c* is the speed of light in vacuum and *a* is the lattice constant, while the *x*-axis plots the coordinates of wavevectors located in the first Brillouin zone (BZ) of the simple cubic lattice. For reference, the inset shows ﻿the location of high-symmetry points in the first BZ used to plot out the bandstructure. Two areas are highlighted which will be analyzed more closely: band 3, lying between *f* = 0.4–0.6, and band 12, located among the higher order bands around *f* = 0.7.

To facilitate this discussion, we plot the iso-frequency surfaces (IFSs) of the photonic dispersion, which map the Bloch wavevectors in k-space that light of a given frequency may occupy. Their significance is that the direction of energy flow for a Bloch wave, given by the group velocity **v**_g_, lies normal to these surfaces. Iso-frequency surfaces are helpful for understanding the mechanics of refraction in PCs, provided refraction is defined in terms of the propagation of energy in accordance with **v**_g_ for non-homogenous media^15^. Figure 2b shows a section of the first BZ with several IFSs plotted for band 3, for frequencies in the range from 0.5 to 0.58. The anisotropic nature of the dispersion is readily discerned from the box-like shape of the IFSs, which exhibit steeply sloped sidewalls running nearly parallel with the *z*-direction. This anisotropy is the origin of parallel-to-interface refraction, as the following example will show.

Consider a cross section of Fig. 2b, taken along the plane *k*_*y*_ = 0, which is shown in Fig. 2c. To see how light is refracted, suppose that the PC/air interface lies in the plane *z* = 0, with the PC occupying the space *z* > 0, and let there be a plane wave with wave vector ${{\bf{k}}}_{0}=\langle {{\rm{k}}}_{{\rm{x}}},0,{{\rm{k}}}_{{\rm{z}}}\rangle$ incident on the PC from air (two such waves are drawn over the iso-frequency curves of Fig. 2c). The geometry of the problem is presented in Fig. 2d. The wave vector **k** of the refracted wave is determined by boundary conditions at the interface, namely: conservation of frequency, and conservation of *k*_*x*_, the component of **k**_0_ that is tangential to the interface. The first boundary condition means that the coordinates of **k** will be found on the IFS whose frequency is the same as that of the incident wave, and the second boundary condition guarantees that the *x*-component of **k** is *k*_*x*_. Referring to Fig. 2c, we consider light with *f* = 0.55, incident from air at an angle θ_0_ = 17° as measured from the *z*-axis, so that *k*_*x*_ = 0.16(2π/*a*); this is marked with a dashed line. The coordinates of **k** (marked with an **×**) are found where this dashed line intersects the IFS having *f* = 0.55. Finally, the direction of **v**_g_, denoted by a large arrow, is given by the normal to the IFS. For this example, the angle between **v**_g_ and the PC/air interface halfway up the sidewall is less than 1°; i.e., the refracted wave travels nearly parallel to the interface. We find a similar response for light with *f* = 0.50 at *k*_*x*_ = 0.26(2π/*a*), or θ_0_ ≈ 31°. The same argument follows for any other ISF in this band. It is noted from Fig. 2c that the dispersion is asymmetric between the *x-* and *z-*directions, despite the fact that the vertical and horizontal lattice constants are the same. The reason for this lies in the lack of a 90°-rotational symmetry in the stacked, layer-by-layer PC configuration.

Band 3 has a simple photonic dispersion that helps elucidate the concept of PIR. This effect may be viewed as a resonance, since it is excited only when **k** lies on the sidewall of an IFS. Accordingly, PIR is strongly wavelength selective. For broadband light-harvesting it is desirable to set up a continuum of densely packed resonances extending over a broad spectral range. This is possible in the higher order bands, which appear in greater density, provided PIR can be found there. Band 12, shown in Fig. 2e,f, satisfies this requirement; its IFSs between 0.69 ≤ 0.73 have straight side walls, and extend over a large section of the BZ, indicating there will be a continuous grouping of abrupt PIR resonances in this wavelength range. It will thus be instructive to investigate how PIR contributes to path length enhancement in a real simple cubic PC.

### Absorption Enhancement in Simple Cubic Photonic Crystal

**Figure 3:**
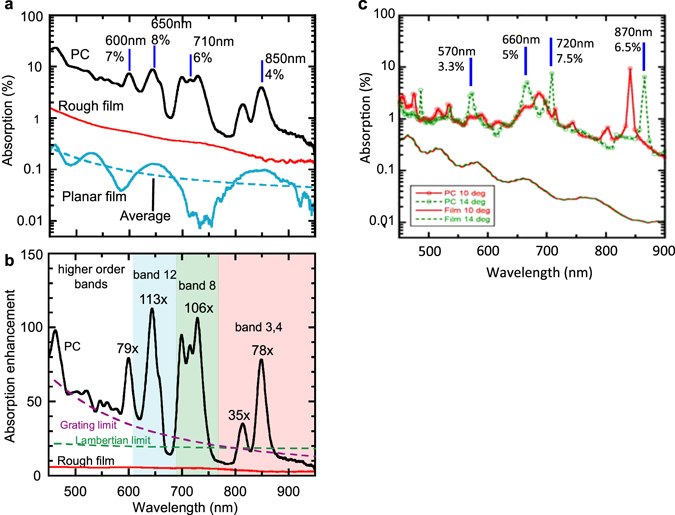
Broadband absorption enhancement in a TiO_2_ photonic crystal. (**a**) Absorption spectrum for PC (black), and reference films R2 (red) and R1 (blue). (**b**) Absorption Enhancement for PC (black) and R2 (red) relative to R1. Also shown are theoretical limits for absorption enhancement in an ergodic system (Lambertian Limit), and in the diffraction grating on absorbing layer scheme. (**c**) An FDTD calculation of the photonic crystal absorption is shown for the wavelength range from 450–900 nm for incident angles of 10° and 14°. Absorption peaks are identified with a blue line.

To investigate PIR-induced path length enhancement, we measure the absorption spectrum of our 4-layer TiO_2_ PC on a fused silica substrate, which is plotted in Fig. 3a for an incident angle of 18°. Two reference samples, designated R1 and R2, are also measured; they are fabricated by depositing TiO_2_ film on top of planar and randomly roughened fused silica substrates, respectively. Detailed information on the reference samples can be found in the Methods section. Figure 3a shows that the absorption of R1 varies between 0.3% and ~0.01%, exhibiting Fabry-Perot oscillations about an average value (dotted line) that decays exponentially, in good agreement with Urbach’s rule for absorption below the semiconductor band edge^32^. R2 shows similar qualitative behavior, except that the oscillations are largely mitigated and absorption increases by 3–5.5 times. However, in the range from 600–800 nm, the PC shows a series of absorption peaks that approach 10%, while the average absorption for R1 is <0.1%. Also, a pair of peaks observed for the PC between 800–900 nm with max values of ~2–4%; here R1 shows 0.05% absorption, while that of the randomly roughened film R2 approaches one tenth of one percent.

To check the legitimacy of these results, a finite-difference time-domain (FDTD) calculation of the absorption in the 4-layer PC was performed for shallow angles of incidence. The results are shown in Fig. 3c and are compared with simulations of the planar reference film. The calculation shows several absorption peaks in the range from 400–900 nm superimposed on a background that varies between 0–2%. In the regime between 660–900 nm, the background absorption from the calculation agrees well with experimental results for the PC, going from ≈0.5% at 900 nm to approximately 1% at 700 nm. Additionally, the location and amplitude of the absorption peaks match well with observations, with the addition of a broadening in the case of the PC, from a full-width half-maximum of 10–30 nm up to 30 or more than 50 nm. In contrast, the lower-wavelength regime brings disagreement between experiment and theory. Below 650 nm the PC background absorption exceeds 5%, and between 450–500 nm the absorption is between 10–20%, compared to 1–2% for the FDTD results. Since the absorption in this region is an order of magnitude higher than what theory predicts, it is apparent that this performance cannot be attributed solely to light-trapping in the PC. One explanation for this discrepancy is possible contamination introduced to the TiO_2_ during fabrication of the PC (see Supplementary Figure S2 in supporting information).

We note that, while the 4% absorption of the PC in the long wavelength range is indeed small, this result is striking, considering that TiO_2_ absorption in this region is usually considered to be negligible. This implies tremendous light trapping capability of the PC due to PIR. To quantify this capability, we denote absorption in the PC and R1 by A_PC_ and A_R1_, respectively, and introduce an enhancement factor η_PC_, defined as the ratio of the PC and R1 absorption values: η_PC_ = A_PC_/A_R1_. For A_R1_, we use the averaged value (dotted line in Fig. 3a). The as-deposited film R1 is most suitable for use as a control, since there is almost no scattering present and the path length is well defined. A similar enhancement η_R2_ is defined for R2. Figure 3b, which plots η_PC_ and η_R2_ as a function of wavelength, more effectively demonstrates the PC’s performance; here, we see that the doublet of peaks observed at longer wavelengths corresponds to η_PC_ = 35 (greater than η_R2_ by 9 times) and 78 (greater than η_R2_ by 24 times). The maximum enhancement for peaks in the mid-wavelength region exceeds one hundred times (>20 times η_R2_). Alternatively, the off-peak enhancement at longer wavelengths fails to exceed the ergodic limit, though it still exceeds the enhancement of R2 by two or three times. This result is likely attributable to random scattering induced by structural disorder in the photonic crystal, the scale of which is estimated from Fig. 1 to be between 30 and 40 nm.

Also shown for comparison are two theoretical limits for enhancement based on different light trapping configurations. One is the ergodic limit of 4*n* ^2^, based on perfect Lambertian scattering from the roughened surface of an absorbing bulk^23^, and the other is the limit for a two-dimensional grating structure as derived in ref. 24 (see Methods section). The various peaks of the PC absorption far surpass both limits, outperforming them by as much as 4–5 times without a back-reflector. The ability of the PC to exceed these limits may be understood from the following argument: for light approaching at some incident angle, surface texturing scatters the light into random directions (random roughening) or various diffracted orders, each with some efficiency (gratings). This will cause the optical path length to be extended for some fraction of the incident intensity, while the remainder experiences forward scattering and marginal enhancement. For a PC, the modes of propagation are determined by the dispersion relation and are uniquely defined, meaning incident light that couples to a PIR resonance all travels in the same direction, with its energy flow pointed nearly parallel to the PC/air interface. This explains why the enhancement peaks of the PC spectrum can exceed the aforementioned limits by multiple times. We will now demonstrate that this enhancement is indeed attributable to PIR resonances.

**Figure 4:**
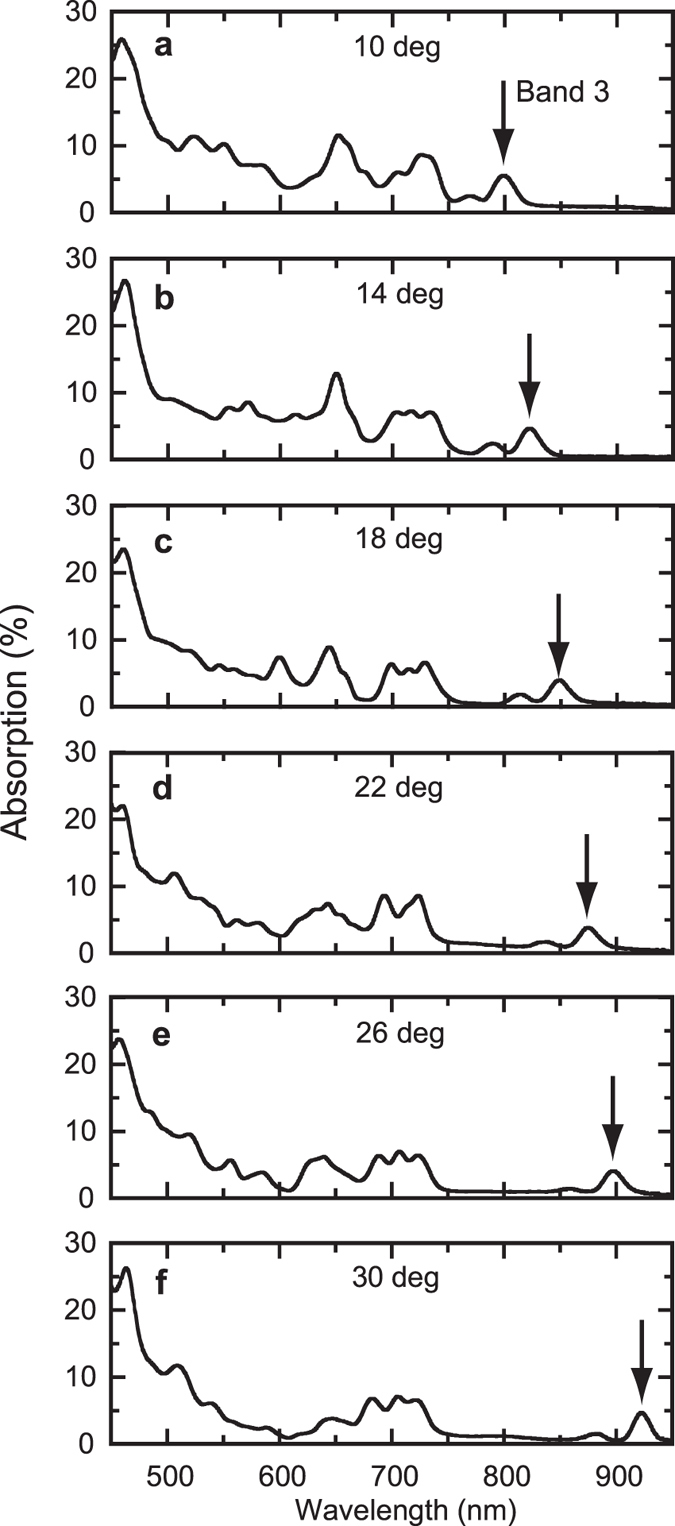
Angular dependence of photonic crystal absorption. (**a–f**) Absorption spectra of TiO_2_ PC for incident angles of 10, 14, 18, 22, 26, and 30 degrees, respectively. An arrow shows the location of the band 3 absorption resonance in each case.

**Figure 5:**
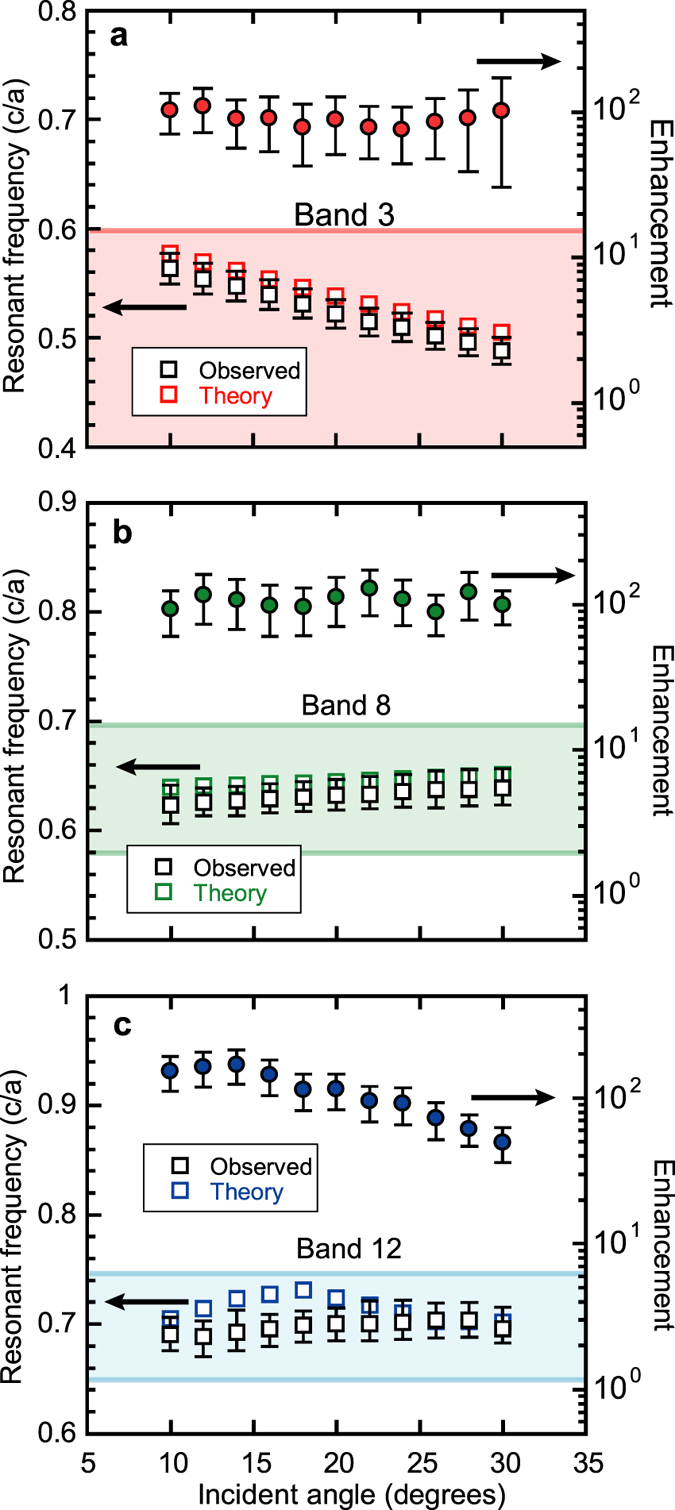
Absorption peak frequency and peak enhancement vs. incident angle. Locations of peak absorption enhancement in the frequency domain as a function of incident angle for (**a**) band 3, (**b**) band 8 and (**c**) band 12. Filled circles are measured enhancement at the peak frequency. Bounded boxes show the frequency range of each band. Enhancement error bars are determined from uncertainty in the reference film absorption measurement. Resonant frequency error bars account for statistical variance in the location of the resonant frequency, uncertainty in identifying an absorption peak with a particular band, and the fact that structural disorder in the PC may create additional absorption peaks that are not predicted by theory (see ref. 13).

The resonant behavior of the PC absorption peaks is most easily demonstrated for bands 3 and 4. To accomplish this, we measure the dependence of the absorption spectrum on the incident angle θ_0_, as shown in Fig. 4. The measured absorption enhancement has also been averaged over incident angles of 10, 12, 14, 16, 18, 20, 22, 24, 26, 28, and 30°, and is discussed in the supplementary information (see supplementary Figure S3). In Fig. 4, the band 3 absorption peak, denoted by an arrow, redshifts from 799 to 922 nm as θ_0_ is increased from 10 to 30°, respectively. Analysis of the IFSs of Fig. 2c shows that this is the expected outcome; due to the negative dispersion of bands 3 and 4, lower-frequency surfaces are found in the outermost region of the BZ, and vice versa. Therefore, longer wavelength PIR resonances will be found at larger incident angles, in agreement with observation. This result can be interpreted another way: for a particular frequency, say, *f* = 0.55, and at some incident angle ${{\rm{\theta }}^{\prime} }_{0}$, *k*_*x*_ is such that **k** is found on the sidewall of the IFS, and PIR results (see Fig. 2c). For ${{\rm{\theta }}}_{0} < {{\rm{\theta }}^{\prime} }_{0}$, *k*_*x*_ is smaller, and **k** lies “on top” of the IFS; **v**_g_ then points along the PC stacking direction, allowing easy transmission. When ${\theta }_{0} > {\theta ^{\prime} }_{0}$, the line marking *k*_*x*_ no longer intersects the IFS and there exists no **k** that satisfies the necessary boundary conditions, resulting in total reflection. Thus, as θ_0_ is swept from low to high values, **k** is scanned across the BZ. The absorption is low, except when θ_0_ sweeps through ${{\rm{\theta }}^{\prime} }_{0}$, where it abruptly increases due to PIR, and then decreases as *k*_*x*_ falls off the IFS. Interpreted this way, the resonances of Fig. 4 yield information about the IFSs of our PC. That is, Fig. 4 constitutes a direct scanning over the photonic dispersion of a real, simple cubic photonic crystal. However, to demonstrate PIR unequivocally, we must confirm that the locations of the observed resonances as a function of θ_0_ agree with those predicted from the IFSs. This is done in Fig. 5 for bands 3, 8, and 12. The peak enhancement vs. θ_0_ is also plotted for each band.

As seen, the observed resonant frequencies not only agree well with the predicted PIR frequencies from theory, but there is a one-to-one correspondence between theory and observation and the scaling of the resonant frequency with θ_0_ is virtually identical. Even band 12 gives reasonable agreement, despite the higher order bands being more sensitive to fabrication error. For bands 3 and 8, η_PC_ at resonance is approximately constant at ~100, while for band 12, η_PC_ peaks exceed 150 for small angles, but steadily decline for θ_0_ > 15°, reaching a value of 49 at θ_0_ = 30°. This is consistent with the band 12 IFSs, which show less inclination with respect to the *x*-axis for *k*_*x*_ > 0.20(2π/*a*), or θ_0_ ≥ 15° (see Fig. 2f).

At this point, we have demonstrated that our PC exhibits absorption peaks with multiple orders-of-magnitude enhancement, that these peaks are resonant in behavior, and that their location and dependence on θ_0_ confirms theoretical predictions made for PIR. The data show that this scanning over the photonic dispersion experimentally validates the concept of parallel-to-interface refraction and provides direct proof of its contribution to absorption enhancement in a simple cubic photonic crystal. As an aside, we mention that slow-light modes are also responsible for absorption enhancement in certain parts of the spectrum. Detailed remarks on the relative importance of this effect and PIR are presented in a supplementary discussion.

## Conclusion

We fabricated a simple cubic PC made from transparent TiO_2_ and used the weak absorption of the reference film to observe broadband, broad-angle absorption enhancement reaching multiple orders of magnitude. Performing a simple angular dependence measurement of this enhancement allowed us to scan the photonic dispersion of our PC and prove unequivocally the presence of parallel-to-interface refraction. This mechanism, in tandem with slow-group velocity effects, should allow the creation of devices whose light trapping capability exceeds the fundamental limits of architectures based on surface scattering in certain frequency ranges. We emphasize that this mechanism is independent of material choice and fabrication method because it results directly from the simple cubic symmetry of the PC. This fundamental principle could be useful for advancing photon harvesting capability in future optoelectronic or photovoltaic applications, such as in phosphor-converted light-emitting diodes, and thin-film solar cells.

## Methods

### Fabrication of TiO_2_ Photonic Crystal

The PC was constructed through a series of deposition, patterning and etch processes using a layer-by-layer method. TiO_2_ was deposited by electron beam evaporation and patterned, using a Cr mask, into a grating with a pitch of 450 nm through deep-ultraviolet lithography and dry-etching in an Oxford reactive-ion etcher with a mixture of CF_4_ and Ar. A lattice constant of 450 nm was chosen so as to place the desired absorptive response in the visible-infrared spectrum, accessible to experimental apparatus. A TiO_2_ layer thickness of 225 nm (=450/2) was chosen so that the lattice constant along the stacking direction, two layers in thickness, would equal the horizontal lattice spacing and a simple cubic structure would result. SiO_2_ was deposited for use as a filler with plasma-enhanced chemical vapor deposition, and excess SiO_2_ was removed with chemical-mechanical polishing. This also made the layer planar in preparation for further deposition of TiO_2_. This process was repeated, rotating the orientation of the TiO_2_ grating by 90° for each layer, until a 4-layer high stack was achieved. Finally, after annealing in O_2_ at 400 °C to bolster TiO_2_ etch resistance, the SiO_2_ was removed with a 5-minute dip in 10:1 buffered-oxide etch (BOE) solution.

### Calculation of Bandstructure and Iso-Frequency Surfaces

The bandstructure was computed using the free MIT Photonic-Bands Package (http://ab-initio.mit.edu/wiki/index.php/MPB). The calculation assumes an infinite simple cubic lattice with a TiO_2_ refractive index of 2.25 and a filling fraction of 50%. Iso-frequency surfaces were computed from the dispersion data.

### Preparation of Reference Samples

TiO_2_ was deposited by electron-beam evaporation under the same conditions used to deposit the TiO_2_ for the photonic crystal. These conditions were as follows: TiO_2_ tablets (99.99% purity) were purchased from International Advanced Materials and deposited at a pressure of ~1 × 10^−5^ Torr to a thickness of approximately 225 nm (the thickness of a layer of the PC). This constitutes 1 deposition. Further depositions were used to increase the thickness of the reference sample. Because no oxygen flow was used during deposition, fresh tablets were melted and used for each deposition. This is also true for the PC. Samples R1 and R2, constructed simultaneously, each consisted of 3 depositions, with a total thickness of 704 ± 17 nm. The thickness of the reference is chosen to be comparable with the total height of the PC (≈850 nm). Attempts to deposit thicker layers resulted in stress-induced cracking and delamination in R1. R1 and R2 were both deposited on double side polished fused silica substrates 1 mm thick, which were diced into square pieces 25 mm on a side. For R2, prior to TiO_2_ deposition, a protective photoresist layer was spun on the backside, and the front side was subjected to bead blasting with corundum abrasive until the substrate became opaque, with a root-mean-square roughness of 4.5 μm. The roughness of R2 is not optimized for absorption or anti-reflection; it was prepared primarily to eliminate oscillations in the absorption spectrum. The substrate was submerged in tetramethylammonium hydroxide at 60 °C for 10 minutes to strip the photoresist, then sonicated in DI water for 5 minutes. Finally, a 5-minute dip in 10:1 BOE was performed to clear the surface of contamination.

### Absorption Measurements

Absorption measurements were preformed with an integrating sphere (Lapsphere) setup using a fiber optic connected vis-IR spectrometer (Ocean Optics USB2000). A tungsten-halogen lamp was used to measure polarization-averaged absorption in the wavelength range from 450–950 nm. All absorption measurements were performed with the sample located inside the sphere. The samples were diced into 2.5 cm × 2.5 cm square pieces and mounted with a Labsphere standard-issue variable angle clamp. The sphere diameter is 6″ (15.24 cm), with a dectector port 0.5″ (1.27 cm) in diameter connected through an ocean-optics fiber optic out-coupler to the spectrometer. The entry port is circular with a 0.5″ diameter. Excluding the extra area provided to the shpere-wall by the interior baffles, the gross area of the integrating sphere-wall is ≈ 729.7 cm^2^. With the subtraction of the entry and exit ports, the net area is ≈ 727.1 cm^2^ and the ratio of the sample area to the net sphere-wall area is ≈ 0.86%. Background spectrum measurements were performed with the sample positioned inside the chamber, but out of the incident beam’s path, to mitigate substitution error. PC measurements were performed using an average of 1500 scans with an integration time of 600 ms. The PC orientation was such that the TiO_2_ bars of the top (surface) layer were perpendicular to the incident plane. Incident angles ranged from 10–30°. Normal incidence measurements were avoided to prevent specular reflection from escaping through the entry port. The PC absorption in Fig. 3 was taken at 18°. Absorption of samples R1 and R2 was measured at an incident angle of 55° in order to baffle the illuminated sections of the chamber wall from the detector field of view for both reflected and transmitted light. The sample mount also has an obstruction at the bottom of the holder to block the illuminated part of the sample from the detector’s field of view. Spectrum captures averaged 100 scans with an integration time of 480 ms. A total of 20 captures were performed and averaged to obtain the final absorption data. The background spectrum was retaken after each capture.

### Rectification of Systematic Error in Reference Sample Measurements

Even in samples where no or very weak absorption is present, the presence of a partially reflective surface in the sphere chamber causes the total optical power falling on the detector to be different than if no sample is present. This is a perturbation in the signal throughput of the integrating sphere, normally on the order of 0.1%. Thus, for the reference samples, a portion of the raw data for λ > 550 nm showed ‘negative’ absorption (see Supplementary Figure S1). The reported data for R1 and R2 uses a scheme for rectifying this error, which is discussed in detail in the supplementary material.

### Calculation of The Theoretical Limits of Figure 3b

The refractive index of e-beam deposited TiO_2_ was measured previously with a Woollam ellipsometer in the wavelength range from 430–1000 nm in 10 nm steps. We measured a refractive index of *n* = 2.24 at λ = 550 nm. The “Lambertian limit” in Fig. 3b was calculated as 4*n* ^2^ using the measured index data. For the “2D grating limit,” we followed the methodology of ref. 24, and assumed a two-dimensional asymmetric square grating with lattice constant ‘*L*’. Under these conditions, the wavelength dependence of the enhancement limit at normal incidence for λ > *L* is 4π*n* ^2^(*L/*λ)^2^. For Fig. 3b, we set *L* = 450 nm.

## Electronic supplementary material

Supplementary Material
