# Supplementary material for: Effectively infinite optical path-length created using a simple cubic photonic crystal for extreme light trapping
Source: Sci Rep. 2017 Jun 23;7:4171. doi: 10.1038/s41598-017-03800-y (PMC5482830; doi:10.1038/s41598-017-03800-y)
Supplement: Supplementary file 1 — Supplementary Material [file 41598_2017_3800_MOESM1_ESM.pdf]

"Effectively infinite optical path-length created using a simple cubic photonic crystal for extreme light trapping"

Brian J. Frey[1], Ping Kuang[1], Mei-Li Hsieh[2], Jian-Hua Jiang[3], Sajeev John[4] and Shawn-Yu Lin[1,*]

[1]*Department of Physics, Rensselaer Polytechnic Institute, 110 8th St., Troy NY 12180*

[2]*Department of Photonics, National Chiao-Tung University, No. 1001, Daxue Rd, East District, Hsinchu City, Taiwan 300*

[3]*School of Physical Science and Technology, Soochow University, 1st Shizi Street, Suzhou, Jiangsu, 215006, China*

[4]*Department of Physics, University of Toronto, 60 Saint George St., Toronto, Ontario M5S 1A7, Canada*

*Correspondence to sylin@rpi.edu

SUPPLEMENTARY MATERIALS

1. Rectification of systematic error in reference sample absorption measurements

As mentioned in the Methods section, raw absorption data for the reference samples R1 and R2 dropped below 0% for wavelengths > 550 nm. In order to draw any conclusions regarding absorption enhancement, it is necessary to rectify this error and demonstrate that the reported data for R1 and R2 is indeed reliable. We will now describe this process and give justification for its use.

In an integrating sphere (IS) direct absorption measurement, the sample is placed inside the chamber, with light incident on it through an aperture on the chamber wall. Light reflects and transmits through the sample, then is diffusely reflected multiple times off of the IS interior (which is covering with a specialized reflective coating) before being collected at the detector. The collected optical intensity is the cumulative reflected and transmitted intensity of the sample. We can write this as

$$\frac{S}{S_0} = T + R, \quad (1)$$

where S is the detected spectral intensity, S_0 is the incident spectral intensity, and T and R are the transmission and reflection coefficients, respectively, with the frequency dependence of these terms being implied. Using the energy conservation condition ($A + T + R = 1$), the absorption coefficient A can be expressed as

$$A = 1 - \frac{S}{S_0}. \quad (2)$$

In the absence of systematic error, the difference of S and S_0 is strictly due to absorption by the sample. However, for real measurements there is a contribution to S, independent of absorption, which arises when a partially reflective sample is placed in the incident beam path. A likely reason for this error is that the sample being illuminated effectively alters the spatially averaged reflectance of the chamber wall, on which the detected photon flux is known to depend. For weakly absorbing samples, this contribution can cause S to exceed S_0 so that A becomes negative. Supplementary Figure S1 shows how this error affects the absorption measurements for R1 and R2. The effect is particularly pronounced for R2

(Supplementary Fig. S1.a), where the absorption for longer wavelengths drops below zero by as much as 0.3 %. The lower bound for R1 (Supplementary Fig. 1.b)

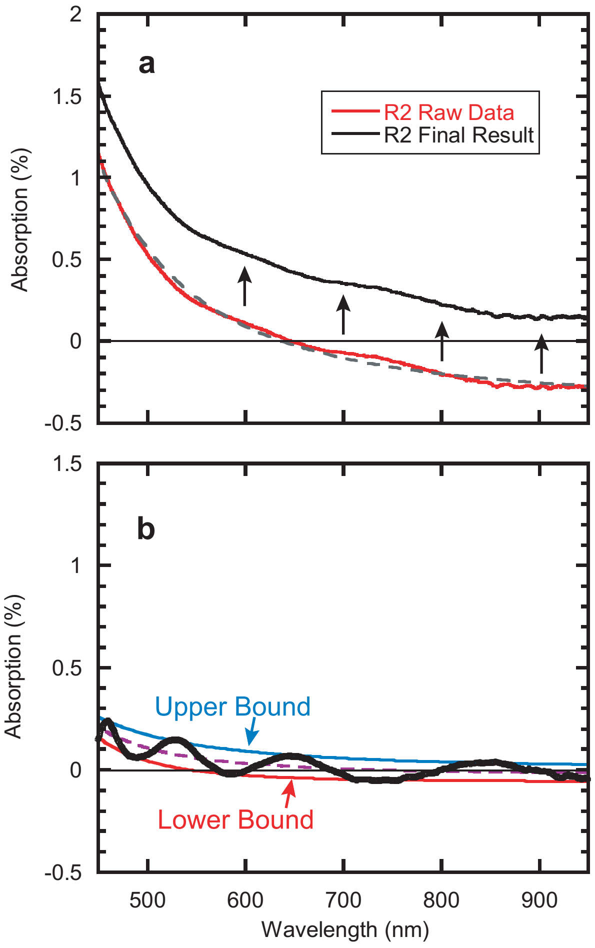

Supplementary Figure S1: Measured absorption data for reference samples R1 and R2. (a) Absorption data for R2 (red) fitted using an exponential function with offset, and same data with the offset removed (black). (b) Absorption data for R1 with upper (blue) and lower (red) bounds of the envelope shown. The average (dotted line) is an exponential function with offset ε_{av}.

reaches ≈ -0.05 %. Let us include this error, denoted by E, in our calculation by transforming S as $S \rightarrow S' = S + E$. We will refer to the 'A' that appears in Equation (2) as A_{ideal}, the measured absorption with E = 0. Then we have

$$A = 1 - \frac{S+E}{S_0} = A_{ideal} - \frac{E}{S_0} \quad . \quad (3)$$

Since E is small compared to S_0, we write $E = \varepsilon S_0$, with $\varepsilon \ll 1$, so that the absorption becomes

$$A = A_{ideal} - \varepsilon. \quad (4)$$

A reasonable first approximation is to assume that ε is wavelength independent in the region of interest. Then, modeling A_{ideal} as an Urbach absorption tail gives us a fit for the data of the form

$$A(\lambda) = C_1 e^{C_2/\lambda} - \varepsilon \ , (5)$$

with C_1, C_2 and the offset ε as constants. For R2, with absorption measured as a percentage, this model fits the data satisfactorily with $\varepsilon = 0.43 \pm 0.05$ (dotted line in Supplementary Fig. S1.a). The final, reported data is obtained by removing this offset from the raw data (black line in Supplementary Fig. S1.a)

The situation is more complicated for R1. There are oscillations in the data, but the upper and lower bounds of the envelope can be fit with Equation 5 to high precision, as seen in Supplementary Figure S1.b. The problem is that the offset derived for the upper bound ε_{high} (= $9.7\times10^{-3} \pm 2.0\times10^{-3}$) is much less than that of the lower bound (ε_{low} = 0.062 ± 0.002), meaning our first approximation for ε is insufficient to describe the behavior of R1. This becomes apparent if we calculate the average of the two bounds (the dotted line of Supplementary Fig S1.b), for which the 'offset' is ε_{av} = 0.036. Simply shifting the R1 data by ε_{av} does not bring the lower bound above zero. However, shifting by ε_{low} provides a good upper limit for the absorption, and this is what we have done to obtain the final result for the R1 absorption plotted in Figure 3 of the text.

2. Experimental investigation of absorption in processed TiO_2 films:

During fabrication of the PC, the sample was exposed to different processing conditions. To determine how these affected the intrinsic absorption coefficient of TiO_2, an experimental procedure was conducted as follows to create a processed reference sample, hereafter called R3:

1.) Deposit TiO_2 by e-beam evaporation according to the fabrication procedure outlined in the Methods section, and in ref. 33 of the manuscript.

2.) Deposition of Cr by sputter deposition, thickness ≈ 40 nm.

3.) Spin-coating of photoresist, and lithography step to pattern 1D grating in photoresist. The line pitch is 450 nm.

4.) ICP-reactive-ion-etching of Cr layer in Trion etcher, using photoresist as a mask for the Cr. Transfer of 1D pattern into Cr layer, exposing TiO_2 surface.

5.) Reactive-ion-etching of TiO_2 using Cr as the mask. The etch recipe is the same as that used to fabricate the PC in ref. 33.

6.) Removal of Cr layer (and liftoff of remaining photoresist) with industrial Cr etchant (Cr Etchant 1020AC, Transene), followed by rinse in DI water.

7.) Repeat step 1.

8.) Planarize layer with chemical-mechanical polishing. Slurry used is Semisperse 12E (same components as previous procedure).

9.) Anneal films for 1 hour at 400° C in O_2 environment.

10.) Dip in 10:1 buffered-oxide etch solution for 1 minute. (PC was dipped to remove SiO_2 template).

11.) Measure the optical absorption of the planarized TiO_2 film and compare with that reported for the reference sample R1.

Results:

The final thickness of the processed films was ≈400 nm. Following step 8, the average height variation was ≈3 nm over a lattice length of 450 nm. Yet, a diffraction pattern was observed, indicating the presence of a remaining 1D grating. This is attributable to air gaps in the TiO_2 layer caused by incomplete filling occurring during step 7. Optical absorption measurements produced a spectrum that qualitatively matched the Ürbach profile of a thin absorbing film, except for the appearance of super-imposed peaks, which had an amplitude of ~0.5-1.0 %. It was not clear if these peaks were the result of diffraction or process contamination.

However, there was a separate area of the same sample, for which the fabrication procedure was identical, except that no grating was produced because the making layer was not patterned in step 3. The absorption of this sample is shown in Supplementary Figure S2 and compared with the reference as-deposited film R1. In the region from 600-900 nm, the data are in good agreement. For wavelengths below 600 nm, the absorption tail of the processed sample begins to rise above that of the un-patterned R1, to a value of approximately 1.5 % at 400 nm; here R1 is oscillating about a value of ≈0.5 %.

Energy-dispersive X-ray spectroscopy (EDS) measurements were performed to check for process residues. It could not detect any signal from chromium on R3 following step 6, although for the patterned region, peaks were observed for carbon

and fluorine, at ≈10 % by weight on the surface, which were attributed to the TiO_2 etch in step 5.

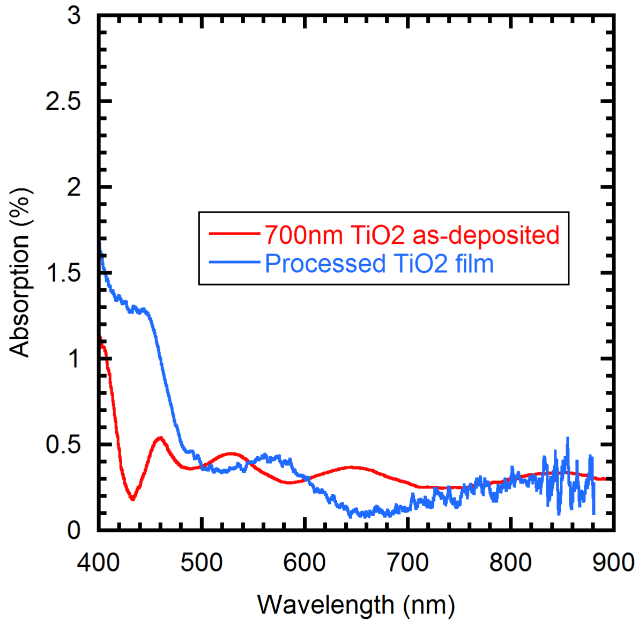

Supplementary Figure S2: Comparison of absorption in as-deposited and processed TiO_2 films. The as-deposited film (red) is a reference film (called R1 in the main text of the manuscript). The thickness of the processed film is ~400 nm. Both sets of data are shifted up by 0.3% for visual clarity.

Based on these observations and the available computational data, we conclude that:

1.) In the range from 650-900 nm, the fabrication process contributes no appreciable effect on material absorption. The background absorption reported in this range is corroborated by simulation, and claims of 100 times enhancement made in the main text are valid.

2.) For wavelengths below 600 nm, high absorption of 5-20% in Figure 3.a of the main text is not supported by FDTD simulations. Processed TiO_2 films show

increased rates of absorption at lower wavelengths. Therefore, the enhancement in this region is not strictly due to light trapping performance of the PC, and processing conditions have introduced some contamination.

2. Analysis of enhancement due to light-bending

Let us explore the relative importance of parallel-to-interface refraction and slow-light effects as regards absorption in the simple cubic PC. According to the Beer-Lambert law, absorption depends on the product $\alpha\ell$ of the absorption coefficient α and the geometric path length ℓ. The PC enhances this product by a factor of $\eta_\alpha \eta_\ell$, where $\eta_\alpha = \alpha_{PC}/\alpha_{film}$ and $\eta_\ell = \ell_{PC}/\ell_{film}$ are the enhancement factors for α and ℓ, respectively. Now, η_ℓ is easily understood as resulting from PC refraction, while η_α arises from the prolonged light-matter interaction of slow-light modes. To wit: $\alpha \propto 1/v_g$, so we have*

$$\eta_\alpha = \rho \frac{(c/n)}{v_g} \text{ , (6)}$$

where *c* is the speed of light in vacuum, *n* is the refractive index of bulk TiO_2 and ρ is the fraction of the optical intensity for a particular mode that is located within the TiO_2 region of the PC. Since the exact value of η_α isn't critical for this discussion we will simplify the analysis by assuming an upper limit with $\rho = 1$. From this, it is evident that both PIR and slow light can contribute to light trapping. Yet, these mechanisms operate independently and it's possible to evaluate their relative

* Mortensen, N. A. & Xiao, S. Slow-light enhancement of Beer-Lambert-Bouguer absorption. *Appl. Phys. Lett.* **90,** 141108 (2007).

importance. More importantly, for TiO_2 we have $\alpha\ell \ll 1$ so that the absorption is linear in $\alpha\ell$. This means a direct correspondence exists between $\alpha\ell$ and the observed absorption. That is,

$$\eta_{PC} \cong \left(\frac{1 - R_{PC}}{1 - R_{film}} \right) \eta_{\alpha} \eta_{\ell} \ . \ (7)$$

The term in parentheses is a weighting factor that accounts for the front-side reflectance R_{PC} and R_{film} of the PC and reference film, respectively. It's wavelength dependence is dominated by R_{PC}, but, barring any directional stop-gaps in the bandstructure where $R_{PC} \approx 1$, we can expect the PC and film reflectance to be within an order of magnitude of each other, and so this weighting factor will be a number on the order of unity. Therefore, if we obtain a theoretical value for v_g from the IFSs and calculate η_{α} from Equation 6, we can extract from our experimental data an order-of-magnitude estimate for η_{ℓ} according to

$$\eta_{\ell} \sim \frac{v_g}{(c/n)} \eta_{PC} \ . \ (8)$$

This is the enhancement of the path length that light experiences as it is refracted by the photonic crystal. Let us first look at the near-infrared absorption peaks between 800-925 nm (Figure 4 of main text) as an example. Here, the full-width at half-maximum Γ of the resonance is about 23 nm and the absorption drops steeply away from the peak frequency. We might deduce from this that η_{PC} for longer wavelengths is driven primarily by PIR. We can determine v_g graphically from the IFSs by measuring the normal distance in *k*-space between two surfaces with frequencies ω and $\omega + \Delta\omega$ in the limit

$$v_g = \left(\lim_{\Delta\omega \to 0} \frac{\Delta k}{\Delta\omega} \right)^{-1}. \quad (9)$$

For example, on the sidewall of the $f = 0.522$ IFS in band 3, $v_g = 0.55c$. Our bulk TiO_2 has $n \approx 2.0$ in this wavelength range, so the speed of light in the film is $0.5c$. We see then from Equation 6 that η_α is less than unity. This means light in this frequency range actually travels a bit faster through the PC than through the film, which hinders the overall absorption enhancement. Nevertheless, Figure 5 shows that, for $\theta_0 = 20°$ there is a peak at $f = 0.522$ belonging to band 3, where $\eta_\ell \sim \eta_{PC} = 88$. This result lends credence to our earlier observation. With the rest of band 3 behaving similarly, we conclude that absorption enhancement in the lower bands is due entirely to PIR.

In contrast, at $\theta_0 = 20°$ for $f = 0.645$ in band 8, Figure 5 shows $\eta_{PC} = 111$. However, now $v_g \approx 0.05c$ and the effect of slow light is much stronger, leading to $\eta_\alpha = 10.9$ and $\eta_\ell = 10.2$. Hence, the effects of slow light and PIR are comparable in band 8. Calculations for Band 12 are hampered by the fact that refraction in this frequency range cannot be attributed to a single band with certainty due to strong band overlap, but the shape of the calculated IFSs, the enhancement being > 100 times, and the good agreement for lower bands leads us to conclude that PIR is present and contributes strongly to the observed results.

In general, slow light becomes more prevalent in the flatter, higher-order bands. PIR can theoretically be found in any band, and this might lead one to think that the more densely grouped high-order bands are the most promising for creating densely packed PIR resonances over a large bandwidth. In reality, the finite

size of the PC introduces an uncertainty to the wavevector, which places limitations on the strength of this effect in the more intricate higher-order bands. This means the dominant contributions to enhancement from PIR are likely to be found for $f < 1$.

3. Averaged absorption enhancement over incident angle

The measured absorption spectra were averaged over incident angles of 10, 12, 14, 16, 18, 20, 22, 24, 26, 28, and 30°. The results are shown in Supplementary Figure S3, where the angle-averaged enhancement is compared with the ergodic limit over the spectral range of 450-950 nm.

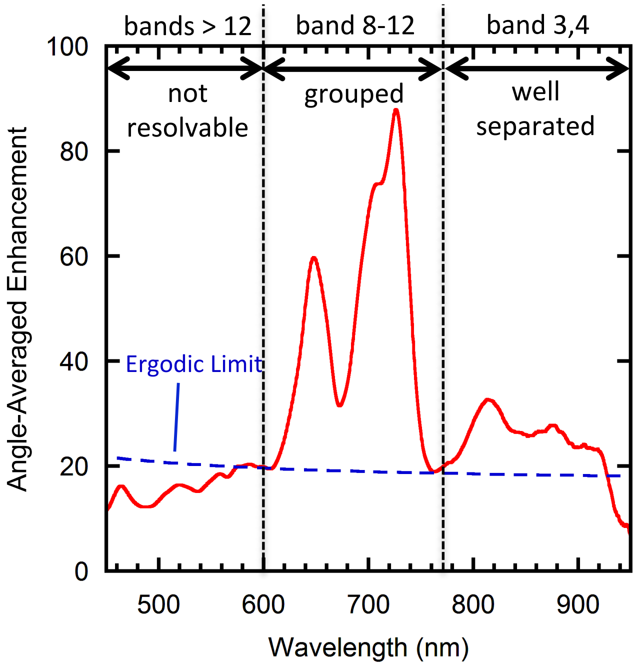

Supplementary Figure S3: Angle-Averaged Enhancement Plot for the 4-Layer TiO_2 Photonic Crystal. The data are averaged over angles of 10, 12, 14, 16, 18, 20, 22, 24, 26, 28, and 30°. The ergodic limit (dotted line) is shown for comparison.

This plot can be divided into three regions: In the region below 600 nm, corresponding to *f* > 0.75, there is significant spectral overlap in the higher order bands. Therefore, absorption peaks cannot be resolved in this region (see Figure 2.a of the main text).

The region between 600-775 nm contains bands 8-12; there is moderate overlap of bands, but a group of absorption peaks can still be resolved as standing out from the background. Additionally, these bands are flatter, in that they cover a smaller frequency range than the lowest order bands, which can also be seen from the dispersion (Figure 2.a). This means that the peaks will shift through a smaller range of wavelengths as θ_0 is varied from low to high angles (as can be seen be comparing Figure 5.a and 5.b of the main text). Consequently, the enhancement in this region has limited angular dependence. In contrast, the lower order bands are more isolated in frequency, allowing PIR peaks to be clearly resolved; they exhibit angular dependence and are wavelength selective, which allows the dispersion to be mapped as discussed for Figure 4. Yet, in the longer wavelength region, in particular for band 3 and 4, the averaged enhancement is still above the ergodic limit. This is because (a) at each angle there is a peak doublet whose maxima are separated by 30-40 nm in wavelength, and (b) each peak is broadened to a FWHM of at least 25 nm.
